# Nurses’ Experiences of their Ethical Responsibilities during Coronavirus Outbreaks: A Scoping Review

**DOI:** 10.1177/08445621221080153

**Published:** 2022-02-15

**Authors:** Elizabeth Peter, Caroline Variath, Shan Mohammed, Mikaela Mitchell, Tieghan Killackey, Jane Maciver, Conor Chiasson

**Affiliations:** 1 Lawrence S. Bloomberg Faculty of Nursing, Joint Centre for Bioethics, 7938 University of Toronto, Toronto, ON, Canada; 2 Lawrence S. Bloomberg Faculty of Nursing, 7938 University of Toronto, Toronto, Canada; 3 Faculty of Health Sciences and Wellness, 10025 Humber College, Toronto, Canada; 4 Gerstein Science Information Centre, 7938 University of Toronto, Toronto, Canada; 5 7979 Hospital for Sick Children, Toronto, Canada

**Keywords:** Ethics, nurses, COVID-19, care, feminist ethics, review

## Abstract

Globally, nurses have experienced changes to the moral conditions of their work during coronavirus outbreaks. To identify the challenges and sources of support in nurses’ efforts to meet their ethical responsibilities during SARS, MERS, and COVID-19 outbreaks a scoping review design was chosen. A search was conducted for eligible studies in Ovid MEDLINE, Ovid Embase and Embase Classic, EBSCO CINAHL Plus, OVID APA PsycInfo, ProQuest ASSIA, and ProQuest Sociological Abstracts on August 19, 2020 and November 9, 2020. The PRISMA-ScR checklist was used to ensure rigor. A total of 5204 records were identified of which 41 studies were included. Three themes were identified related challenges in meeting ethical responsibilities: 1) substandard care, 2) impeded relationships, 3) organizational and system responses and six themes relating to sources of support: 1) team and supervisor relationships, 2) organizational change leading to improved patient care, 3) speaking out, 4) finding meaning, 5) responses by patients and the public, 6) self-care strategies.Our review revealed how substandard care and public health measures resulted in nurses not being fully able to meet their ethical responsibilities of care. These included the visitation policies that impeded the support of patients by nurses and families, particularly with respect to face-to-face relationships. Organizational and system responses to the evolving outbreaks, such as inadequate staffing, also contributed to these challenges. Supportive relationships with colleagues and supervisors, however, were very beneficial, along with positive responses from patients and the public

## Background

Currently 90% of National Nursing Associations have reported that they are concerned with heavy workloads, a lack of resources, and increasing numbers of nurses experiencing stress and burnout as a result of caring for patients during the COVID-19 pandemic (International Council of Nurses [ICN], 2021a). Several literature reviews have described the severe psychological impacts on healthcare workers during recent epidemics and pandemics (Carmassi et al., 2020; Preti et al., 2020; Shaukat et al., 2020). Joo and Liu (2021) have also conducted a literature review that has revealed the barriers that nurses have encountered when caring for COVID-19 patients related to limited and constantly changing information, unpredictable responsibilities, a lack of support, concerns about the safety of their own families, and psychological stress. However, much less attention has been paid to the moral conditions of nurses’ work during the COVID-19 pandemic and previous Severe Acute Respiratory Syndrome (SARS) and Middle East Respiratory Syndrome (MERS) outbreaks. Several recent studies (Iheduru-Anderson, 2021; Lapum et al., 2021; Rezee et al., 2020; Sperling, 2021) have reported nurses’ ethical concerns about shifts in the standard of nursing practice, including patients dying in isolation, and have examined nurses’ viewpoints regarding resource allocation during the COVID-19 pandemic. No literature review to date, however, has synthesized and appraised the growing empirical evidence on how previous coronavirus outbreaks and the current pandemic have impacted the capacity of nurses to meet their ethical responsibilities. In addition, no review of the literature has explored what has helped nurses sustain their efforts in the contexts of major shifts in practice because of COVID-19.

Nurses are morally responsible to their patients and communities: to promote health, to prevent illness, to restore health and to alleviate suffering and promote a dignified death” (ICN, 2021b, p. 2). Theorists, such as Vanlaere and Gastmans (2011), have further delineated what nurses’ ethical responsibilities are using a theoretical lens of *care*. Care involves both an attitude of ‘caring about’ that entails nurses’ emotional and attentive response to patients and ‘caring for’ that requires nurses to take responsibility in engaging in caring activities to meet the needs of their patients often in the context of a face-to-face interaction (Vanlaere & Gastmans, 2011). As an ethical responsibility, care must be other-regarding in that attention must turn to the needs of others (Vanlaere & Gastmans, 2011). From a care perspective, moral emotions are elicited in those who care (Vanlaere & Gastmans, 2011), which have been defined as “those emotions that are linked to the interests or welfare either of society as a whole or at least of persons other than the judge or agent” (Haidt, 2003, p. 853).

Meeting the ethical responsibility to care for others requires that nurses are also cared for and have their needs met so that they can temporarily ignore their own goals and concerns to recognize and attend to the needs of others (Tronto, 1993). Not only are patients dependent on nurses to meet their needs, nurses are also dependent on patients to maintain their moral identity. Through expressing gratitude and displaying improvement in their health or well-being, patients enable nurses to develop a sense that they are good nurses (Peter et al., 2018; Vanlaere & Gastmans, 2011). Ultimately, through caregiving nurses can find life fulfillment and meaning, but with limited close contact with patients, nurses often experience stress because of their compromised abilities to fulfill the responsibilities of care (Vanlaere & Gastmans, 2011).

## Purpose

The purpose of this scoping review was to identify the challenges and sources of support in nurses’ efforts to meet their ethical responsibilities during the SARS, MERS, and COVID-19 outbreaks. We chose to examine the findings of studies conducted involving SARS and MERS, along with those during the COVID-19 pandemic, because nurses’ experiences during these outbreaks may follow similar patterns, they all are the result of potentially lethal coronaviruses with a comparable mode of viral transmission, and each yielded a widespread public health response. The following research questions guided our review:

1. What challenges did nurses experience fulfilling their ethical responsibilities of care during the SARS, MERS, and COVID-19 outbreaks?
2. What fostered nurses’ capacity to fulfill these responsibilities during the SARS, MERS, and COVID-19 outbreaks?

## Methods

### Design

We chose a scoping review to allow a mapping of the broad range and extent of research occurring related to nurses’ responsibilities and moral emotions during the COVID-19 pandemic and previous coronavirus disease outbreaks (Arksey & O; alley, 2005). Specifically, this review was guided by the methodological framework initially proposed by Arksey and O’Malley (2005), and advanced by Levac et al. (2010). The framework consisted of five stages, including identifying research questions, searching for relevant studies, study selection, charting data, and analysing and reporting the data. To improve rigor in the methodology and process, steps outlined by Tricco et al. (2018) in the ‘Preferred Reporting Items for Systematic Reviews and Meta-Analyses Extension for Scoping Reviews (PRISMA-ScR)’ were followed.

### Search methods

To identify relevant articles, we followed the three-step search strategy outlined by the Joanna Briggs Institute (Peters et al., 2020). First, a preliminary search on the topic identified was conducted in two databases (MEDLINE and EMBASE). The search terms included coronavirus (e.g., COVID-19, SARS, MERS), nurse or midwife, moral distress, ethics, morals, psychology, stress, work environment etc. This initial search yielded very few relevant studies and therefore, in collaboration with a health sciences librarian (MM), we broadened the search. The key search terms used included nurses, COVID-19, SARS, and MERS (Table 1).

**Table 1.** Supplemental Resource: Database Search Strategy.

**Database**	**Search Term**	**Results**
**Ovid Medline** **Ovid MEDLINE**: Epub Ahead of Print, In-Process & Other Non-Indexed Citations, Ovid **MEDLINE**® Daily and Ovid **MEDLINE**® <1946-Present>	1. exp Nurses/ 2. exp Nursing Staff/ 3. Licensed Practical Nurses/ 4. nursing/ or evidence-based nursing/ or nursing, practical/ 5. exp Specialties, Nursing/ 6. Students, Nursing/ 7. exp Nursing Care/ 8. exp Nursing Process/ 9. (nurse* or nursing*).tw,kf. 10. (midwif* or midwiv*).tw,kf. 11. personal support worker*.tw,kf. 12. (healthcare aid* or health care aid*).tw,kf. 13. or/1-12 [Nurses] 14. exp Coronavirus Infections/ 15. exp Coronavirus/ 16. (coronavirus* or corona virus* or ncov* or cov or covid*).tw,kf. 17. (mers or middle east respiratory syndrome*).tw,kf. 18. (sars* or severe acute respiratory syndrome*).tw,kf. 19. or/14-18 [COVID-19 + MERS + SARS] 20. 13 and 19	**2160**
**Ovid Embase** **Embase Classic **+** **Embase <1947 to 2020 November 06>	1. exp nurse/ 2. nursing staff/ 3. licensed practical nurse/ 4. nursing/ or cultural nursing/ or evidence based nursing/ or holistic nursing/ or humanistic nursing/ or international nursing/ or practical nursing/ or telenursing/ or travel nursing/ 5. exp nursing assessment/ 6. exp nursing care/ 7. nursing career/ 8. nursing competence/ 9. exp nursing discipline/ 10. nursing expertise/ 11. nursing intervention/ 12. nursing knowledge/ 13. exp nursing management/ 14. nursing outcome/ 15. exp nursing practice/ 16. nursing process/ 17. nursing role/ 18. exp nursing student/ 19. midwife/ 20. nurse attitude/ 21. midwife attitude/ 22. (nurse* or nursing*).tw,kw. 23. (midwif* or midwiv*).tw,kw. 24. personal support worker*.tw,kw. 25. (healthcare aid* or health care aid*).tw,kw. 26. or/1-25 [Nurses] 27. exp coronavirinae/ 28. exp Coronavirus infection/ 29. (coronavirus* or corona virus* or ncov* or cov or covid*).tw,kw. 30. (mers or middle east respiratory syndrome*).tw,kw. 31. (sars* or severe acute respiratory syndrome*).tw,kw. 32. or/27-31[COVID-19 + MERS + SARS] 33. 26 and 32	**2533**
**Ovid PsycInfo** **APA** PsycInfo <1806 to November Week 1 2020>	1. exp nurses/ 2. nursing/ 3. nursing students/ 4. (nurse* or nursing*).tw. 5. (midwif* or midwiv*).tw. 6. personal support worker*.tw. 7. (healthcare aid* or health care aid*).tw. 8. or/1-7 [Nurses] 9. (coronavirus* or corona virus* or ncov* or cov or covid*).tw. 10. (mers or middle east respiratory syndrome*).tw. 11. (sars* or severe acute respiratory syndrome*).tw. 12. or/9-11 [COVID-19 + MERS + SARS] 13. 8 and 12	**145**
**Ebsco CINAHL** Plus with Full Text	S22. S15 AND S21 S21. S16 OR S17 OR S18 OR S19 OR S20 S20. TI ((sars* or severe acute respiratory syndrome*)) OR AB ((sars* or severe acute respiratory syndrome*)) S19. TI ((mers or middle east respiratory syndrome*)) OR AB ((mers or middle east respiratory syndrome*)) S18. TI ((coronavirus* or corona virus* or ncov* or cov or covid*)) OR AB ((coronavirus* or corona virus* or ncov* or cov or covid*)) S17. (MH "Coronavirus Infections + ") S16. (MH "Coronavirus + ") S15. S1 OR S2 OR S3 OR S4 OR S5 OR S6 OR S7 OR S8 OR S9 OR S10 OR S11 OR S12 OR S13 OR S14 S14. TI ((healthcare aid* or health care aid*)) OR AB ((healthcare aid* or health care aid*)) S13. TI personal support worker* OR AB personal support worker* S12. TI ((midwif* or midwiv*)) OR AB ((midwif* or midwiv*)) S11. TI ((nurse* or nursing*)) OR AB ((nurse* or nursing*)) S10. (MH "Students, Nursing + ") OR (MH "Students, Nursing, Practical") S9. (MH "Nursing Role") S8. (MH "Nursing Practice + ") S7. (MH "Nursing Administration + ") S6. (MH "Nursing as a Profession") S5. (MH "Practical Nursing") S4. (MH "Nursing Assessment") S3. (MH "Nursing Care + ") S2. (MH "Nursing Assistants") S1. (MH "Nurses + ")	**2850**
**ProQuest Applied Social Sciences Index & Abstracts (ASSIA)**	((MAINSUBJECT.EXACT.EXPLODE("Nursing") OR MAINSUBJECT.EXACT.EXPLODE("Nurses")) OR noft(nurse* or nursing or midwif* or midwiv* or personal support worker* or healthcare aid* or health care aide*)) AND (MAINSUBJECT.EXACT("SARS") OR noft(coronavirus* or corona virus* or ncov* or cov or covid* or mers or middle east respiratory syndrome* or sars* or severe acute respiratory syndrome*))	**560**
**ProQuest Sociological Abstracts**	((MAINSUBJECT.EXACT("Midwifery") OR MAINSUBJECT.EXACT("Nurses")) OR noft(nurse* or nursing or midwif* or midwiv* or personal support worker* or healthcare aid* or health care aide*)) AND noft(coronavirus* or corona virus* or ncov* or cov or covid* or mers or middle east respiratory syndrome* or sars* or severe acute respiratory syndrome*)	**44**

**Figure 1.**
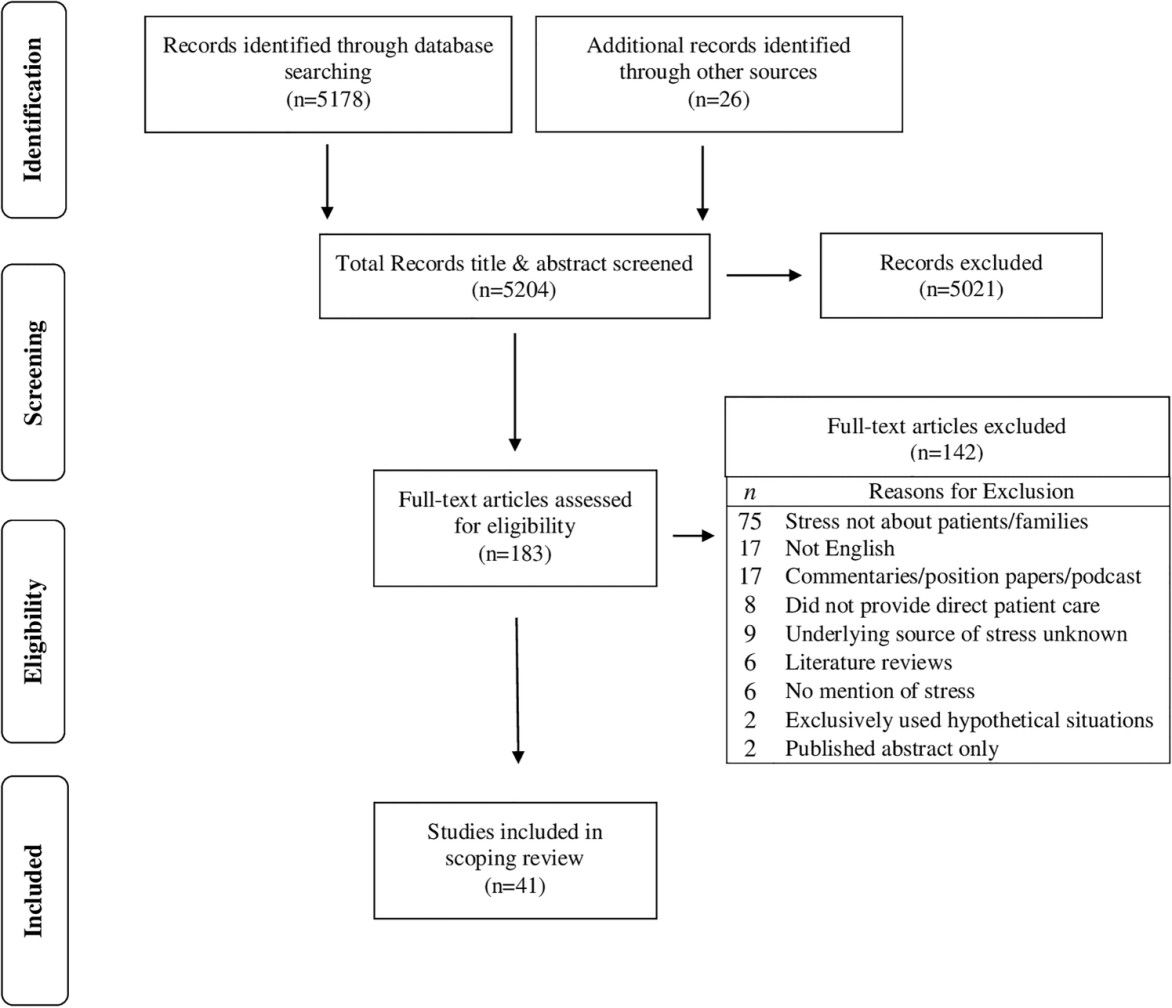
PRISMA flow diagram of study selection process.

Searches were then conducted in the following databases on August 20, 2020 by the health sciences librarian (MM) (Table 1): Ovid MEDLINE (Epub Ahead of Print, In-Process & Other Non-Indexed Citations, Ovid MEDLINE® Daily and Ovid MEDLINE® 1946-Present), Ovid Embase + Embase Classic (1947 to 2020 August 19), EBSCO CINAHL Plus with Full Text (1981 to present), OVID APA PsycInfo (1806 to Present), ProQuest ASSIA, and ProQuest Sociological Abstracts. A combination of database specific subject headings and textwords were used to search for the concepts of nurses, COVID-19, SARS, and MERS as well as relevant synonyms. No limits or filters were applied. A draft of the Ovid Medline search was peer reviewed by a second health sciences librarian using the PRESS guidelines (McGowan et al., 2016). Search results were deduplicated in EndNote™ using the optimized method by Bramer et al. (2016), then uploaded to Covidence™ where remaining duplicates were identified. All searches were updated and re-run on November 9, 2020. New studies were identified using EndNote™, as outlined by Bramer and Bain (2017) and uploaded to Covidence™ for screening. Additionally, the reference list of all the sources that met the inclusion criteria and past literature reviews were hand-searched to identify additional sources (Figure 1). No further searches were conducted for reasons of feasibility.

Initial title and abstract screening were conducted by two team members (EP and CV). The inclusion and exclusion criteria (Table 2) were refined during the early stages of the screening process (Levac et al., 2010). These were chosen to identity empirical studies of nurses or midwives who had provided direct care to patients with SARS, MERS or COVID-19 and had expressed concerns in the form of moral emotions, i.e., stress or distress, which is consistent with Vanlaere and Gastmans (2011)'s articulation of ‘caring about’ which includes attentiveness and emotions expressed for the other. Each article was tagged as 'include', 'maybe' and 'exclude' in Covidence™. Full-text screening of studies marked ‘include' and ‘maybe' were conducted independently by two team members (EP and CV). Discrepancies in the screening process were resolved by rereading articles and collaborating with the team (Levac et al., 2010).

**Table 2.** Inclusion and Exclusion Criteria.

**INCLUSION CRITERIA**	**EXCLUSION CRITERIA**
**Nurses and midwives who provided direct care to patients affected by the outbreak** **Original peer-reviewed research published since 2000** **COVID-19, SARS, MERS outbreaks** **English** **Stress of nurses or midwives**	Research reported in the gray literature and conference abstracts Conceptual and position papers Literature reviews Stress/distress not focused on patients or the public Publications that made only a token reference to nurses’ or midwives’ stress

### Search outcomes

Our review yielded a total of 41studies from 16 countries, including five quantitative and 36 qualitative studies (Figure 1). Of these studies 29 were about the COVID-19 pandemic, 10 about SARS outbreaks, and two about MERS outbreaks. Most studies involved nurses exclusively (29), while others had participants from other healthcare professions and occasionally family members as well (12). When results pertained to another professional group or family members exclusively, we did not include that material in our extractions or findings.

### Quality appraisal

A formal appraisal of reviewed papers is not a requirement for a scoping review (Levac et al., 2010) and therefore, will not be reported.

### Data abstraction

Data abstraction and charting were an iterative process, that allowed the team to become familiar with the sources and provide a summary of the articles included in the study (Peters et al., 2020). Key information about the source of evidence from the included articles was recorded in a tabular form. The extracted information included author(s), year of publication, country of origin, type of coronavirus, aims/purpose, participants, research design, sources of concern and stress, and preventive and supportive elements. (Table 3)

**Table 3.** Abbreviated Extraction Table.

Author (year)	Country	Study type	Purpose	Participants
**Archer et al. (2020)**	United Kingdom	Cross-sectional qualitative survey	To gain perspectives on the impact of COVID-19 on psycho-oncology activity; specifically, on how services, teams and individuals are adapting under the strains of the pandemic	94 participants (nurses, physicians, allied health professionals etc.)
**Ardebili et al. (2020)**	Iran	Qualitative	To undertake an in-depth exploration of the experiences of the mental health consequences of healthcare staff working during the COVID-19 crisis	86 participants (nurses, physicians, emergency services, pharmacists etc.)
**Arnetz et al. (2020)**	United States	Cross-sectional qualitative survey	To explore perceptions of the most salient sources of stress in the early stages of the COVID-19 pandemic in a sample of U.S. nurses	455 nurses (registered nurses and advanced practice registered nurses)
**Azoulay et al. (2020)**	France	Cross-sectional quantitative survey	To assess the prevalence and determinants of symptoms of anxiety, depression, and peritraumatic dissociation in critical care healthcare providers exposed to COVID-19	1058 participants (nurses, physicians, allied health professionals etc.)
**Bahramnezhad and Asgari (2020)**	Iran	Qualitative (phenomenology)	To explain the lived experiences of nurses in the care of patients with COVID-19 to create a comprehensive description of this care and to understand the intrinsic structure of this phenomenon	14 nurses
**Blanco-Donoso et al. (2021)**	Spain	Cross-sectional quantitative survey	To analyze the psychological consequences that this COVID-19 crisis is having on nursing home workers and, as well, the influence that work stressors and inadequate job resources could have on the development of those consequences	228 participants (nurses, social workers, psychologists etc.)
**Bournes and Ferguson-Paré (2005**)	Canada	Qualitative (phenomenology)	To describe the experience of persevering through a difficult time for patients, family members of patients, nurses, and allied health professionals during the SARS outbreak	63 participants (nurses, allied health professionals, patients and family members)
**Butler et al. (2020)**	United States	Qualitative	To describe the perspectives and experiences of clinicians involved in institutional planning for resource limitation and/or patient care during the COVID-19 pandemic	61 participants (nurses, nurse practitioners and physicians)
**Cai et al. (2020)**	China	Cross-sectional quantitative survey	To investigate the psychological impact and coping strategies of frontline medical staff in Hunan province, adjacent to Hubei province, during the COVID-19 outbreak between January and March 2020	534 participants (nurses, physicians and other hospital staff).
**Catania et al. (2021)**	Italy	Qualitative (descriptive)	To explore nursing management issues within COVID-19 narratives of Italian front-line nurses	23 nurses
**Chiang et al. (2007)**	Taiwan	Qualitative (hermeneutics)	To provide an interpretative account of nurses’ self-state, which evolved in response to the demands of treating SARS patients	21 nurses
**Chung et al. (2005)**	Hong Kong	Qualitative (phenomenology)	To explore in depth the experiences of nurses’ caring for SARS patients in Hong Kong	8 nurses
**Digby et al. (2021)**	Australia	Qualitative survey	To determine the impact of working during the early stage of the COVID-19 pandemic on the well-being of staff at one 600-bed acute hospital in metropolitan Melbourne, Australia	321 participants (medical, nursing, allied health and non-clinical staff)
**Fan et al. (2020)**	China	Qualitative (descriptive)	To collect the experiences and views of transdisciplinary nurses at the forefront of the COVID-19 outbreak to evaluate their psychological stresses	25 nurses
**Gagnon and Perron (2020)**	Canada	Qualitative	To gain insights into Canadian nurses’ use of media for sharing their experiences, raising concerns, speaking up, blowing the whistle, and advocating for themselves and their clients during COVID-19	83 news stories reporting nurses’ experiences
**Galehdar et al. (2020)**	Iran	Qualitative	To explore nurses’ experiences of psychological distress during care of patients with COVID-19	20 nurses
**Góes et al. (2020)**	Brazil	Qualitative (descriptive)	To identify the challenges faced by paediatric nursing workers in the face of the COVID-19 pandemic	26 nurses
**Hall et al. (2003)**	Canada	Qualitative	To describe nursing work life issues as portrayed in the media during the SARS crisis in Toronto	35 news stories reporting nurses’ experiences
**He et al. (2021)**	China	Qualitative (phenomenology)	To examine the experiences of Chinese nurses who countermarched to the outbreak city for medical support in the first period of this (COVID-19) global infection	10 nurses
**Hou et al. (2020)**	China	Qualitative (phenomenology)	To explore the preparedness of the emergency department in a tertiary hospital in Taiyuan, Shanxi province, from the nurses’ perspectives during the COVID-19 outbreak	12 nurses
**Iheduru-Anderson (2021)**	United States	Qualitative (phenomenology)	To describe the lived experience of acute care nurses working with limited access to PPE during the COVID-19 pandemic	28 nurses
**Jia et al. (2021)**	China	Qualitative (descriptive)	To examine the ethical challenges encountered by nurses caring for patients with COVID-19 and share their coping styles to ethical conflicts and dilemmas	18 nurses
**Kackin et al. (2021)**	Turkey	Qualitative (phenomenology)	To determine the experiences and psychosocial problems among nurses caring for COVID-19 patients in Turkey	10 nurses
**Karimi et al. (2020)**	Iran	Qualitative (phenomenology)	To explore the lived experiences of nurses caring for patients with COVID-19 in Iran	12 nurses
**Kates et al. (2021)**	United States	Cross-sectional quantitative survey	To understand the impact of the COVID-19 pandemic on the hospice and palliative workforce and service delivery	36 participants (nurses, allied health professionals)
**Kim (2018)**	South Korea	Qualitative (phenomenology)	To identify psychological stress in nurses who cared for MERS patients and to identify systemic problems of the Korean healthcare system	12 nurses
**Koller et al. (2006)**	Canada	Qualitative (ethnography)	To examine the experiences and perspectives of children hospitalized because of SARS, their patients, and pediatric health care providers	23 participants (healthcare providers, children and parents)
**Lee et al. (2020)**	South Korea	Qualitative (phenomenology)	To explore the experiences of Korean nurses who had directly cared for patients with MERS and to derive the structure and meaning of these experiences	17 nurses
**Lee et al. (2005)**	Taiwan	Quantitative survey	To understand the needs and experiences of frontline female nurses in order to provide better psychiatric services in future epidemics. (SARS)	26 nurses
**Leong et al. (2004)**	Singapore	Qualitative	To examine how a palliative care team perceived the psychosocial and spiritual needs that arose as health care workers, patients and their families dealt with SARS	8 participants (nurses, physicians, social workers and pharmacist)
**Liu and Liehr (2009)**	China	Qualitative (descriptive)	To identify instructive messages to guide nursing practice in future epidemics by examining the stories of Chinese nurses who cared for SARS patients	6 nurses
**Liu et al. (2020a)**	China	Qualitative (phenomenology)	To describe the experiences of physicians and nurses caring for COVID-19 in the early stages of the outbreak	13 participants (nurses and physicians)
**Liu et al. (2020b)**	China	Qualitative	To explore the experiences of front-line nurses combating the COVID-19 epidemic	15 nurses
**Sarabia–Cobo et al. (2020)**	Spain, Italy, Peru, Mexico	Qualitative (phenomenology)	To explore the emotional impact and experiences of geriatric nurses working in nursing homes and caring for patients with COVID-19	24 nurses
**Schroeder et al. (2020)**	United States	Qualitative (descriptive)	To explore the experience of being a registered nurse caring for patients with COVID-19 at an urban academic medical center during the early stages of the pandemic	21 nurses
**Sheng et al. (2020)**	China	Qualitative (phenomenology)	To explore the influence of experiences of involvement in the COVID-19 rescue task on professional identity of nurses	14 nurses
**Shih et al. (2007)**	Taiwan	Qualitative	To identify the stage-specific difficulties encountered by Taiwan's surviving frontline nurses during the anti-SARS process	200 nurses
**Shih et al. (2009)**	Taiwan	Qualitative	To explore Taiwan's nurse leaders' reflections and experiences of the difficulties they encountered and survival strategies they employed, while fighting the SARS epidemic and the background context framing these phenomena	70 nurses
**Sun et al. (2020)**	China	Qualitative (phenomenology)	To explore the psychology of nurses caring for COVID-19 patients	20 nurses
**Tan et al. (2020)**	China	Qualitative (phenomenology)	To explore the work experience of clinical first-line nurses treating patients with COVID-19	30 nurses
**Travers et al. (2020)**	United States	Qualitative	To explore the relationship between organizational empowerment structural components and feelings of psychological empowerment among hospital frontline workers during COVID-19	13 nursing assistants

### Synthesis

The abstracted data was then collated and summarized by identifying themes that describe and synthesize key patterns and narratives in the literature (Arksey & O; alley, 2005). To supplement our thematic analysis, we also carefully considered how the data fit the theorization of ethical responsibilities of care to identify both sources of challenge and support in the chosen studies.

## Results

We found three themes related to challenges in meeting ethical responsibilities: 1) substandard care, 2) impeded relationships, 3) organizational and system responses and six themes relating to sources of support that helped nurses to meet their ethical responsibilities: 1) team and supervisor relationships, 2) organizational change leading to improved patient care, 3) speaking out, 4) finding meaning, 5) responses by patients and the public, and 6) self-care strategies.

### Challenges in meeting ethical responsibilities

#### Substandard care

Nurses experienced intense feelings of helplessness and perceptions of futility when caring for patients during coronavirus outbreaks. Nurses expressed concern when the usual standards of care could not be met (Butler et al., 2020; Gagnon & Perron, 2020; Iheduru-Anderson, 2021; Kackin et al., 2021; Schroeder et al., 2020; Shih et al., 2007). Certain studies specified the nature of substandard care, such as when poor infection control measures could have led to the spread of the virus to patients, (Gagnon & Perron, 2020; Iheduru-Anderson, 2021; Kim, 2018), a hospital bed could not be supplied upon admission (Tan et al., 2020), patients did not receive life-saving treatments or adequate attention (Butler et al., 2020; Iheduru-Anderson, 2021; Liu et al., 2020a), COVID screening and time needed to don and doff personal protective equipment (PPE) led to delays in treatment and care (Hou et al., 2020; Jia et al., 2021; Kates et al., 2021), or home visits were limited (Digby et al., 2021; Kackin et al., 2021).

Substandard care related to end of life of care was also reported, such as when end-of-life decision-making occurred too quickly (Azoulay et al., 2020) or end-of-life care was not dignified (Lee et al., 2020). Facilitating the proper treatment of dead bodies and assisting with funerals and other ceremonies related to death and dying were also identified as part of participants’ responsibilities that could not be fulfilled adequately (Galehdar et al., 2020; Leong et al., 2004). Specifically, Iheduru-Anderson (2021) reported how nurses struggled with “the ethics of working below the accepted standard of care” (p. 9). Other studies (Archer et al., 2020; Digby et al., 2021; Jia et al., 2021; Leong et al., 2004) identified the prioritization of COVID-19 patients led to the withdrawal and the restriction of treatment options for non-COVID-19 patients as a source of concern.

Nurses’ reports of substandard patient care because of coronavirus measures were accentuated by the redeployment of nurses to practice areas, such as intensive care, in which they did not have confidence in their abilities (Arnetz et al., 2020; Catania et al., 2021; Digby et al., 2021; He et al., 2021; Jia et al., 2021; Lee et al., 2020; Liu et al., 2020a, 2020b). For instance, Catania et al. (2021) reported how new graduates often became the most senior professionals on COVID units despite their lack of experience.

#### Impeded relationships

Nurses expressed a serious concern that they could not form adequate caring and humanizing relationships with patients given the barriers presented by the need to wear PPE and to limit direct contact (Bahramnezhad & Asgari, 2020; Bournes & Ferguson-Paré, 2005; Butler et al., 2020; Hall et al., 2003; Karimi et al., 2020; Koller et al., 2006; Leong et al., 2004; Sun et al., 2020). For example, Bournes and Ferguson-Paré (2005) spoke of PPE “smothering connectedness” (p. 328) because compassionate facial expressions were difficult to convey as the result of wearing a mask, making the demonstration of compassion and responsiveness, two of nurses’ core values (ICN, 2021b), difficult. Similarly, Leong et al. (2004) described how PPE requirements resulted in the unrecognizability of healthcare professionals and the taboo of touching patients without gloves, bringing about a disruption in connectedness.

Public health measures, which restricted visitors from coming into hospitals, prevented families from supporting their loved ones, especially at end-of-life. Multiple papers described how these restrictions constrained family-centered care and the inability to prevent and reduce patient suffering (Arnetz et al., 2020; Azoulay et al., 2020; Bournes & Ferguson-Paré, 2005; Butler et al., 2020; Chung et al., 2005; Digby et al., 2021; Iheduru-Anderson, 2021; Jia et al., 2021; Kackin et al., 2021; Koller et al., 2006; Lee et al., 2020; Leong et al., 2004; Sheng et al., 2020). For example, Iheduru-Anderson (2021) reported nurses’ “horror of watching people die alone without their loved ones” (p. 7) because they could not be present.

#### Organizational and system responses

Organizational and system responses to the evolving pandemic contributed to contexts in which nurses could not meet their ethical responsibilities. Frequently cited were the shortage of PPE to protect patients (Arnetz et al., 2020; Blanco-Donoso et al., 2021; Karimi et al., 2020), and the lack of equipment (Butler et al., 2020; Karimi et al., 2020; Lee et al., 2005), adequate training (Arnetz et al., 2020; Góes et al., 2020; Karimi et al., 2020; Liu & Liehr, 2009), staff (Ardebili et al., 2020; Blanco-Donoso et al., 2021; Butler et al., 2020; Catania et al., 2021; Gagnon & Perron, 2020; Góes et al., 2020; Kackin et al., 2021; Karimi et al., 2020; Liu et al., 2020a, 2020b; Sarabia–Cobo et al., 2020; Shih et al., 2009; Tan et al., 2020; Travers et al., 2020) and clarity of responsibilities (Fan et al., 2020). Other researchers also reported team dysfunction, including the avoidance of infected patients (Cai et al., 2020; Chiang et al., 2007; Shih et al., 2007), as difficult. With these shortcomings, nurses could not provide an adequate standard of care in the context of an outbreak.

Gagnon & Perron (2020); Iheduru-Anderson (2021), and Karimi et al. (2020) highlighted the problem of ill-prepared local healthcare systems. Gagnon and Perron (2020) used the words “deplorable” (p. 112) and “systemic negligence” (p. 112) to describe the working environment in which nurses experienced inadequate staffing, mandatory overtime, and poor communication while trying to care for COVID-19 patients. In one American study, (Arnetz et al., 2020), nurses experienced constraints in their ability to voice their perspectives regarding public health measures, even while at work, because the pandemic had become so politicized, and nurses who worked through the SARS epidemic in Taiwan criticized the government for the lack of a good SARS response, expressing the importance of their participation as core decision makers in any future health crisis planning at the local and national levels (Shih et al., 2007).

### Sources of support

#### Team and supervisor relationships

The importance of team relationships was identified frequently as essential to the continuation of the capacity to care (Azoulay et al., 2020; Bahramnezhad & Asgari, 2020; Cai et al., 2020; Catania et al., 2021; Chiang et al., 2007; Chung et al., 2005; He et al., 2021; Hou et al., 2020; Jia et al., 2021; Kim, 2018; Lee et al., 2005; Lee et al., 2020; Liu et al., 2020a; Liu & Liehr, 2009; Sheng et al., 2020; Shih et al., 2007; Shih et al., 2009; Sun et al., 2020; Travers et al., 2020). Authors of several studies also identified heightened levels of cooperation and cohesion during the pandemic or outbreak (Bahramnezhad & Asgari, 2020; Catania et al., 2021; Hou et al., 2020; Sun et al., 2020). Chung et al. (2005) emphasized the importance of how the non-hierarchical nature of the team during SARS increased collegiality and team spirit.

Several studies also pointed to the significance of support received from supervisors and leaders (Blanco-Donoso et al., 2021; Digby et al., 2021; Lee et al., 2005; Liu et al., 2020a; Liu & Liehr, 2009; Sheng et al., 2020; Travers et al., 2020). Other authors spoke of the provision of food (Cai et al., 2020; Lee et al., 2005; Liu et al., 2020a), additional education and training, (Jia et al., 2021; Lee et al., 2005) and institutionally provided mental health services (Cai et al., 2020; Lee et al., 2005).

#### Organizational change leading to improved patient care

A small number of studies described how a coronavirus outbreak led to active measures on the behalf of front-line nurses to improve the standard of care such as leading organizational change to improve patient care, (Hou et al., 2020; Jia et al., 2021; Liu & Liehr, 2009; Shih et al., 2007; Travers et al., 2020) including a shift to virtual care (Archer et al., 2020; Digby et al., 2021). Shih et al. (2009) reported that nurses repeatedly asked for additional equipment and more human resources, such as extra staffing, to cope with changes to practice. Jia et al. (2021) described ‘active control and planning’ (p. 7) which led nurses to find ways to raise the standard of care through efforts such as developing nursing specific plans of care and sharing transferable knowledge from patient cases.

#### Speaking out

The Gagnon and Perron (2020) and Hall et al. (2003) studies were unique in that they used news stories to illustrate nurses’ experiences and to recognize the individual and collective voices of nurses. Gagnon and Perron (2020) described how nurses providing direct care expressed their concerns to the media regarding substandard care to the public, while Hall et al. (2003) reported on nurses’ speaking out about visitation policies and nurse leaders’ efforts to make changes to the healthcare system.

### Finding meaning in work

Despite the hardships experienced in their work, nurses spoke of developing strength and resilience by finding meaning and expressing pride in their work (Chiang et al., 2007; Lee et al., 2020; Liu et al., 2020ab; Sheng et al., 2020; Shih et al., 2009; Sun et al., 2020; Tan et al., 2020). More specifically, some spoke of fulfilling their professional duties and oaths (Ardebili et al., 2020), affirming their commitment to God (Ardebili et al., 2020), obtaining a divine sense of purpose (Fan et al., 2020; Shih et al., 2009), and coming to terms with their own mortality (Chiang et al., 2007) as ways of finding meaning.

#### Responses by patients and the public

Nurses found expressions of gratitude and appreciation by patients and their families to be supportive (Bahramnezhad & Asgari, 2020; Chung et al., 2005; He et al., 2021; Kim, 2018; Lee et al., 2020; Liu et al., 2020a, 2020b; Shih et al., 2009; Sun et al., 2020; Tan et al., 2020; Travers et al., 2020) along with witnessing patient improvement (Cai et al., 2020; Chung et al., 2005; Lee et al., 2005; Lee et al., 2020; Liu et al., 2020a, 2020b; Sheng et al., 2020). The satisfaction and appreciation of patients was connected to nurses finding meaning in their work, including a heightened sense of professional identity (Tan et al., 2020).

The expression of public gratitude was viewed as essential (Azoulay et al., 2020; Sheng et al., 2020). Specifically, being viewed as a hero, helped some nurses feel appreciated and valued by the public (Bahramnezhad & Asgari, 2020; Sun et al., 2020). Others also spoke of the importance of the government support and encouragement (Bahramnezhad & Asgari, 2020; Sheng et al., 2020), higher salaries and allowances (Jia et al., 2021; Lee et al., 2005; Sheng et al., 2020; Sun et al., 2020), honorary awards (Sheng et al., 2020; Sun et al., 2020) and career development (Jia et al., 2021; Sun et al., 2020).

#### Self-care strategies

A great variety of self-care strategies were presented in the studies we reviewed. Leisure activities such as reading, watching TV and movies, listening to podcasts (Cai et al., 2020; Iheduru-Anderson, 2021; Kackin et al., 2021; Lee et al., 2005; Liu et al., 2020a; Sun et al., 2020) and spending time on hobbies and recreational activities (Jia et al., 2021; Kackin et al., 2021; Lee et al., 2005) were cited. Other strategies such as obtaining support from family and friends (Cai et al., 2020; Digby et al., 2021; Iheduru-Anderson, 2021; Jia et al., 2021; Lee et al., 2005; Lee et al., 2020; Liu et al., 2020a; Shih et al., 2009; Sun et al., 2020; Travers et al., 2020); venting emotions, e.g. crying (Cai et al., 2020; Iheduru-Anderson, 2021; Jia et al., 2021; Kackin et al., 2021; Lee et al., 2005; Sun et al., 2020); avoiding media about the pandemic/outbreak (Cai et al., 2020; Digby et al., 2021; Kackin et al., 2021; Lee et al., 2005; Liu et al., 2020a); avoiding overtime (Cai et al., 2020; Iheduru-Anderson, 2021); maintaining routines, (Iheduru-Anderson, 2021); seeking help from a personal therapist (Cai et al., 2020); engaging in mindfulness, yoga or prayer, (Digby et al., 2021; Iheduru-Anderson, 2021; Lee et al., 2005; Shih et al., 2009; Sun et al., 2020; Travers et al., 2020); writing (Liu et al., 2020a; Sun et al., 2020); assuming a positive attitude (He et al., 2021; Kackin et al., 2021; Lee et al., 2005; Shih et al., 2009); exercising (Digby et al., 2021; Iheduru-Anderson, 2021; Kackin et al., 2021; Lee et al., 2005; Liu & Liehr, 2009); showering (Liu et al., 2020a); resting and sleeping (Iheduru-Anderson, 2021; Lee et al., 2005; Liu et al., 2020a; Sun et al., 2020); and having a balanced diet (Lee et al., 2005; Liu et al., 2020a; Liu & Liehr, 2009; Sun et al., 2020) were reported to help.

## Discussion

The results of our scoping review revealed numerous challenges to nurses’ efforts to meet their ethical responsibilities during previous coronavirus outbreaks and the COVID-19 pandemic. These were structural in origin because they were related to the lack of clinical, financial, informational, and supportive resources and the impact of public health measures on nursing practice and the standard of nursing care. As healthcare professionals, nurses are responsible and accountable for their nursing practice (ICN, 2021b), yet because nurses working during SARS, MERS, and COVID-19 were often without adequate resources, such as appropriate staffing and equipment, and were working under strict public health measures, they often could not fully meet all their ethical responsibilities of care simultaneously.

The infection control measures, such as visitation policies and PPE, limited the relational work of nurses, especially with respect to face-to-face interactions. The inability of family members to be physically present with patients, particularly at the end of life, was a significant source of concern for nurses. While literature in public health ethics speaks to the ethical tension of balancing the rights of individuals with that of the collective good when making decisions regarding public health measures (Smith & Upshur, 2019), such as visitation policies and decisions to ration healthcare resources during public health emergencies, these issues are discussed assuming that the reader or moral agent is in the position to make these decisions. In this review, however, the nurses did not express concern as a result of decision-making or moral dilemmas, but in contrast, expressed concern as a result of not being able to meet their caring responsibilities in everyday work. Decision-making regarding resources and public health measures had already occurred at a higher level of public health officials and organizational leaders, who may not have had direct links to nurses in frontline practice or understand the forces that compel nurses to ethically respond to those under their direct care.

In their classic work, Lützén et al. (2003) explain how nurses who provide direct care are accountable for the standard of care they provide but they are not often involved in the policies that structure their work. Moreover, they argue that policies generally reflect utilitarian principles that are oriented to maximizing benefit for broader groups and populations, while nurses generally are focused on individual patients’ needs and are acutely aware of their vulnerability and best interests. The results of our study are aligned with the analysis of Lützén et al. (2003) because the impact of organizations on nurses’ day-to-day work came to the forefront as problematic for nurses with respect to their caring efforts. We argue that the intended ethical dimensions of policies, such as infection control and staffing measures, often do not reflect or take into consideration nurses direct moral experiences of these policies and the implications of these measures on their work in close proximity to patients.

We also observed that many (75) publications that we deemed eligible for full-text review were excluded because there was no clear evidence that nurses were experiencing moral emotions or could not fullfil their ethical responsibilities related to their concerns regarding patients and patient care. Instead, the emotions described in these papers were mainly about nurses’ anxiety of becoming infected themselves or infecting their family members. This excluded group of the literature may not have employed data collection techniques that elicited responses about patient care concerns or nurses in these studies, despite the difficult working conditions, were able to provide good nursing care. Alternatively, these studies may reflect nurses’ inability to experience moral emotions and put their own needs aside temporarily when their own health and safety needs are not being met. In essence, it is possible that these nurses were not adequately cared for themselves to be able to be attentive to and care for others adequately.

A variety of self-care strategies were reported including receiving support from family and friends, along with practices related to mindfulness, including meditation, prayer, and yoga. These strategies are relevant to nurses’ caring capacity because nurses require care themselves to provide care for others (Vanlaere & Gastmans, 2011), and they can help nurses build moral resilience in the face of moral distress (Rushton, 2016). Along with self-care strategies, nurses found that their relationships with the healthcare team, especially nurse colleagues, to be very helpful. Other work by Traudt et al. (2016) has also found that the presence of a moral community, in which nurses can openly discuss and gain support with their moral concerns, can be an excellent source of support for nurses to continue to practice ethically. Finding meaning in their work and receiving the responses of patients and the public were also identified by many reviewed papers as supportive which is in keeping with the work of Vanlaere and Gastmans (2011) and Peter et al. (2018) who have described the importance of patients’ and the public's reactions in sustaining nurses’ moral identities as people who care for others and can make a difference in their lives. It may also be that these experiences help nurses to reflect on their moral responsibilities in a pandemic from a broader perspective, helping to place their work into a context that recognizes the realistic expectations for patient care during the constraints of a public health emergency.

Our scoping review did not find many studies that showed nurses’ efforts to directly address the underlying challenges they encountered in their efforts to meet their ethical responsibilities of care, such as speaking out about their working conditions or engaging in political advocacy. Only eight studies (Archer et al., 2020; Digby et al., 2021; Hou et al., 2020; Jia et al., 2021; Liu & Liehr, 2009; Shih et al., 2007; Shih et al., 2009; Travers et al., 2020) demonstrated ways in which nurses influenced organizations to improve the standard of care, and only one study (Hall et al., 2003) reported that staff nurses voiced their concerns regarding visitation policies. These types of active responses are critical because they can directly influence or eliminate sources of these challenges, such as substandard care and impeded relationships. It is possible that these efforts did exist, but the nature of the research studies could not capture this data. Future inquiry might examine nurses’ opportunities to change the conditions of their work to fulfill their moral responsibilities and whether organizations are more open to hearing their voices.

It is essential to recognize that substandard care not only has an immediate deleterious impact on patients, it also erodes the moral identity of nurses in such a way that it could have a cascading impact on their ability to provide ethical care for future patients. As the COVID-19 pandemic continues, and other future health crises emerge, the need for a sustainable nursing workforce that can meet its moral responsibilities is apparent. Governments and administrators plan and provide care environments that make it possible for nurses to offer ethically good care.

### Limitations

This scoping review included studies that had been published up until November 2020. As a result, it did not capture many studies related to nursing ethics during the COVID-19 pandemic that have been published since that time. Moreover, because most of the studies we examined did not mention ethics explicitly, we needed to infer the ethical struggles that these nurses encountered. In addition, in some instances, participants in the reviewed studies included healthcare providers other than nurses which may have slightly impacted our findings.

## Conclusions

Our review revealed how common challenges such as substandard care, as well as unique ones related to public health measures, resulted in nurses not being fully able to meet their ethical responsibilities of care. The former included organizational and system responses to the evolving outbreaks, such as inadequate staffing, and the latter included the visitation policies and the barriers presented by PPE which impeded the support of patients by nurses and families, particularly with respect to face-to-face relationships. These findings point to the direct impact of public health policies across multiple areas of nursing practice, including acute care and long-term care. The need for healthcare organizations, which are often tasked with translating broader public health policies into local practice requirements, to formally involve front-line nurses in this process is essential to promote transparency, accountability, and opportunities for feedback. Nurses providing direct care also need to be included in decisions regarding the prioritization of patients, as they have insight into how these decisions will impact direct patient care.
